# Supplementary material for: Operando two-terminal devices inside a transmission electron microscope
Source: Commun Eng. 2023 Nov 23;2:83. doi: 10.1038/s44172-023-00133-9 (PMC10956025; doi:10.1038/s44172-023-00133-9)
Supplement: Supplementary file 2 — Supplementary Information [file 44172_2023_133_MOESM2_ESM.pdf]

Supplementary information:

Title: Operando Two-terminal Devices inside a Transmission Electron Microscope

Authors: Oscar Recalde-Benitez[1], Tianshu Jiang[1], Robert Winkler[1], Yating Ruan[2], Alexander Zintler[1], Esmaeil Adabifiroozjaei[1], Alexey Arzumanov[2], William A. Hubbard[4], Tijn van Omme[3], Yevheniy Pivak[3], Hector H. Perez-Garza[3], B.C. Regan[4,5], Lambert Alff[2], Philipp Komissinskiy[2] and Leopoldo Molina-Luna[1*]

Affiliations:

[1]Advanced Electron Microscopy Division, Institute of Materials Science, Department of Materials-and Geosciences, Technische Universität Darmstadt; Darmstadt, Germany.

[2]Advanced Thin Film Technology Division, Institute of Materials Science, Department of Materials-and Geosciences, Technische Universität Darmstadt; Darmstadt, Germany.

[3]DENSsolutions; Delft, The Netherlands

[4]NanoElectronic Imaging, Inc., Los Angeles, CA, USA

[5]University of California, Los Angeles and the California NanoSystem Institute, Los Angeles, USA.

*Corresponding author Email: leopoldo.molina-luna@aem.tu-darmstadt.de

MIM Device*	Top electrode	Intermediate layer	Bottom electrode
**Memristor (a1)**	Pt	$SrTiO_3$	Nb-doped STO
**Memristor (a2)**	Pt	Sm-doped CeO_2:STO	Nb-doped STO
**Varactor (b1)**	Au/Pt	$BaSrTiO_3$ (BST)	$SrMoO_3$ (SMO)
**Varactor (b2)**	Au/Pt	Mn-doped BST	SMO

Table S1. Thin-film, two-terminal MIM oxide devices studied in this work. *A sketch of each MIM device is shown in Fig. S11, including the thicknesses of each layer in the corresponding stack.

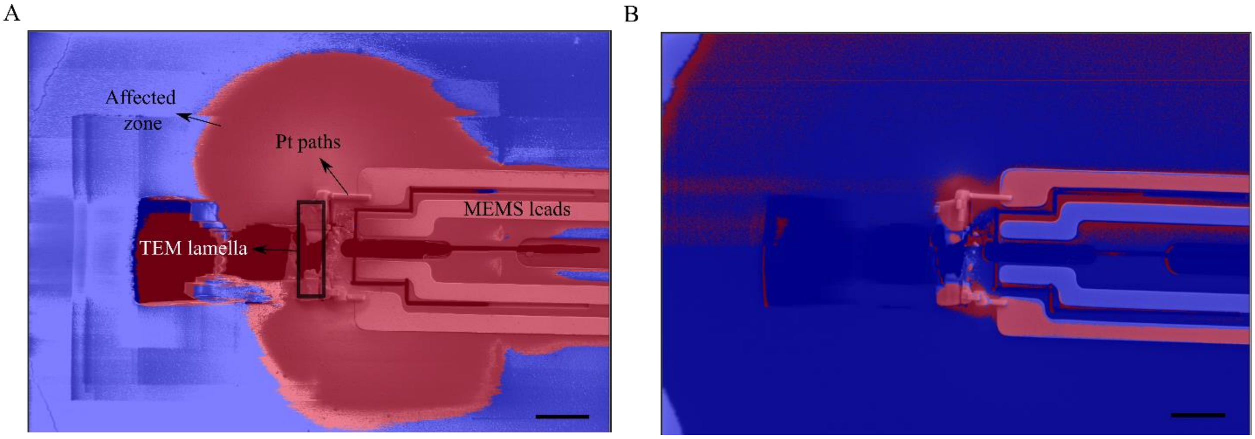

Fig. S1. SEM-Electron beam induced current (EBIC) images showing the affected zone by using GIS-FIB deposited contacting material on a MEMS-based chip. SEM-EBIC images of a MEMS-based chip of a TEM *lamella* linked with Pt. The red contrast in the images represents the affected zone (charging and short-circuited area) across the MEMS-based chip and the *lamella* itself. (**A**) The EBIC signal was acquired at low energy voltages (2kV) to have surface sensitivity (smaller electron penetration depth is expected at this energy). Note how the MEMS leads are short-circuited between them. (**B**) SEM-EBIC image taken at 20kV, as expected, the larger penetration depth conceals the surface contamination due to Pt contamination and charging effects. The scale bar is 10 μm.

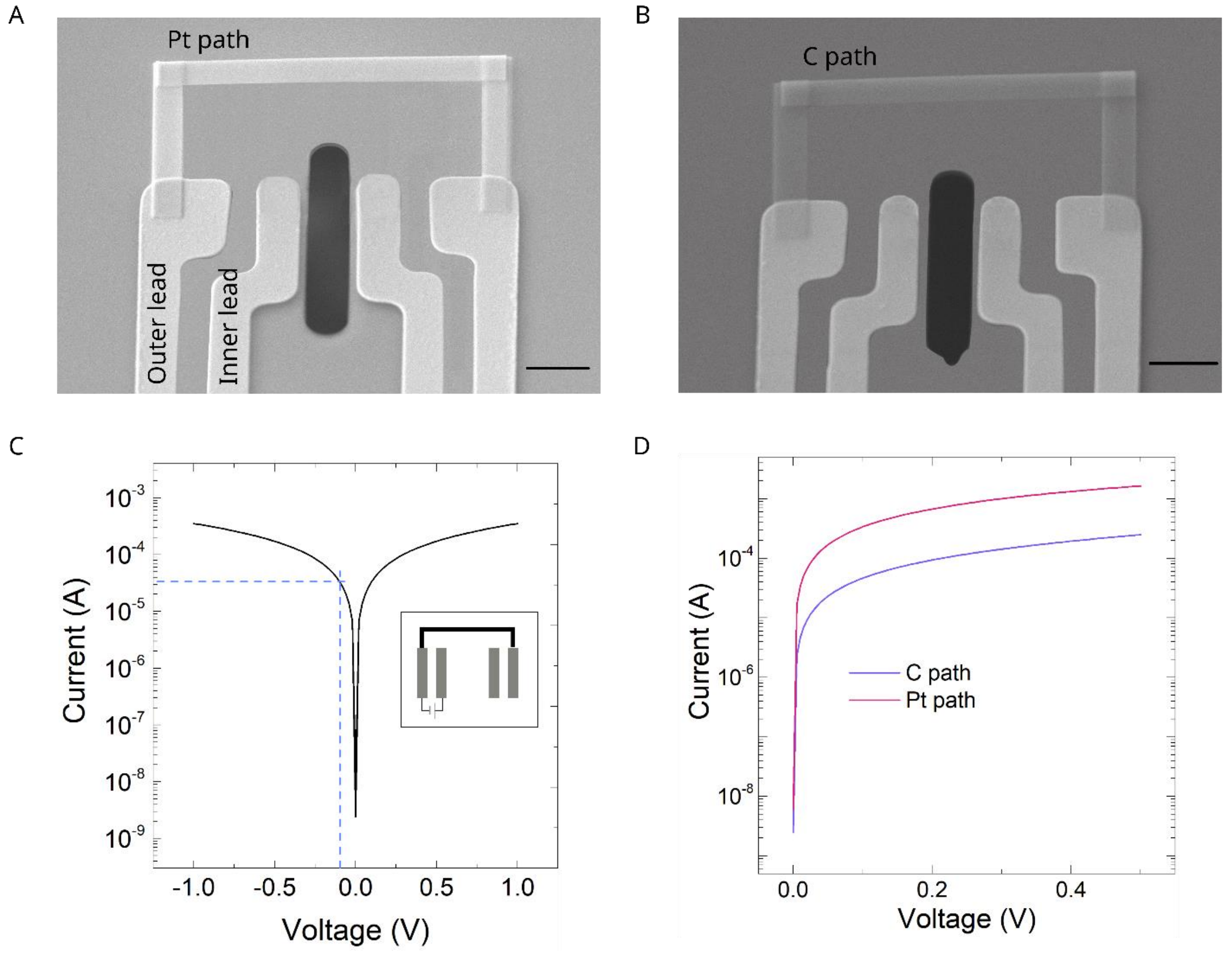

Fig. S2. FIB-deposited pathways in an empty MEMS-based chip (without TEM *lamella*). (**A**) SEM view of 200 nm thickness Pt-path linking the outer leads. (**B**) SEM view of 200 nm thickness C-path linking the outer leads. (**C**) I-V curve obtained from the inner and outer electrodes as shown in the image inset. Note that at 0.1 V the current acquired is in the range of 10^{-5} A (blue dash line), showing that Pt/C contamination is spread between the leads forming electrical links between them. (**D**) I-V curves between outer leads of Pt (red curve) and C (purple curve) paths as show in (A) and (B). The currents achieved for either the C or Pt at 0.1 V are in the range of 10^{-5} A, same current levels obtained for the contamination shown in (C). The scale bar in 5 µm.

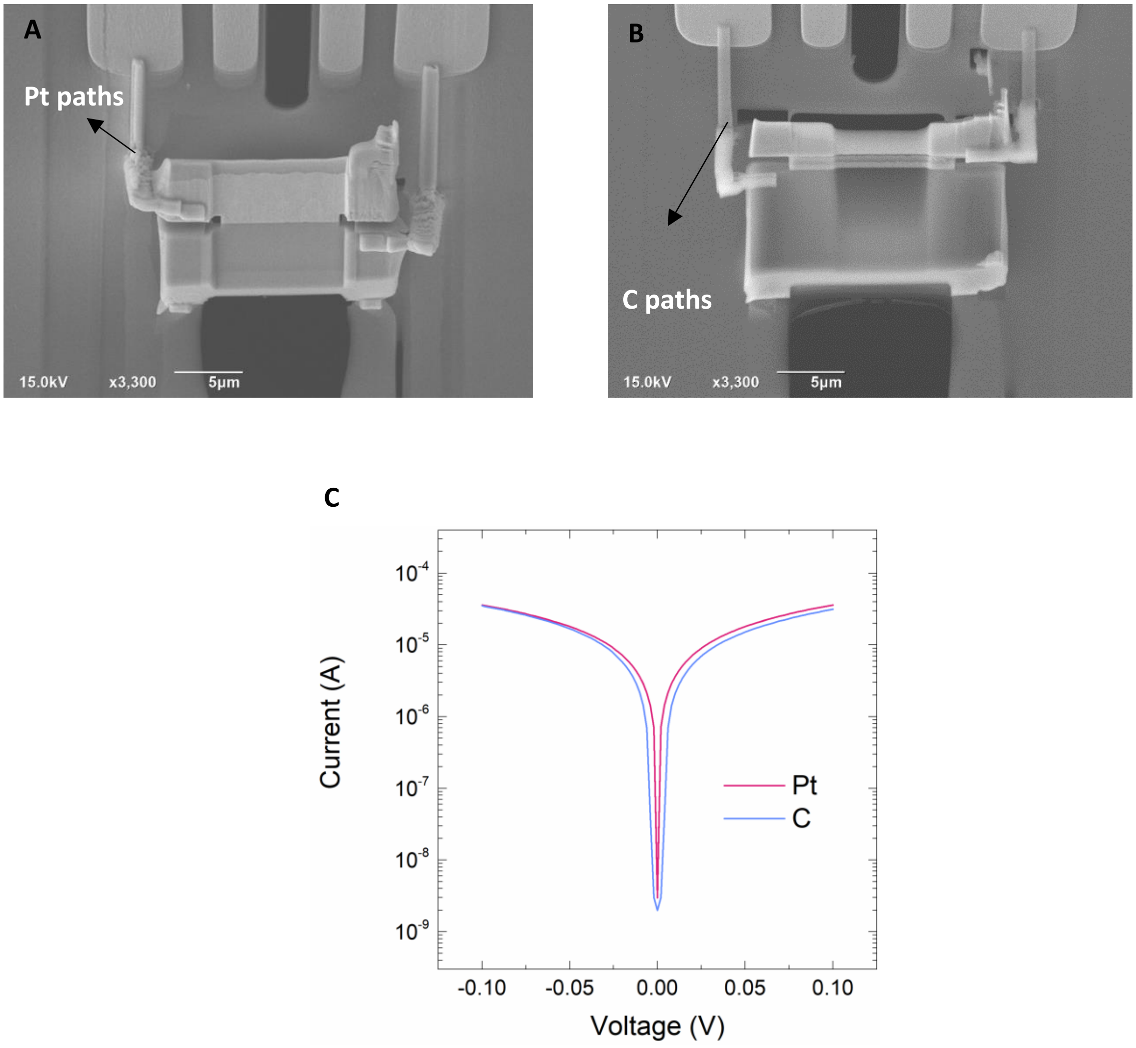

Fig. S3. TEM *lamella* devices linked by the GIS-FIB approach. (**A**) TEM sample device of memristor a1 electrically linked with Pt-paths. (**B**) TEM *lamella* device of memristor a1 linked with C-paths. In general, the samples are placed in the MEMS-based chip with a tilt angle that allows the thinning process on the chip. The Pt or C paths are FIB-deposited during the thinning procedure. Horizontal cuts above and below the contacts are performed to split the bottom and top electrodes to avoid short-circuits. (**C**) Current levels achieved by this method. Note at 0.1 V the current level is similar to the short-circuited version shown in Fig. S2.

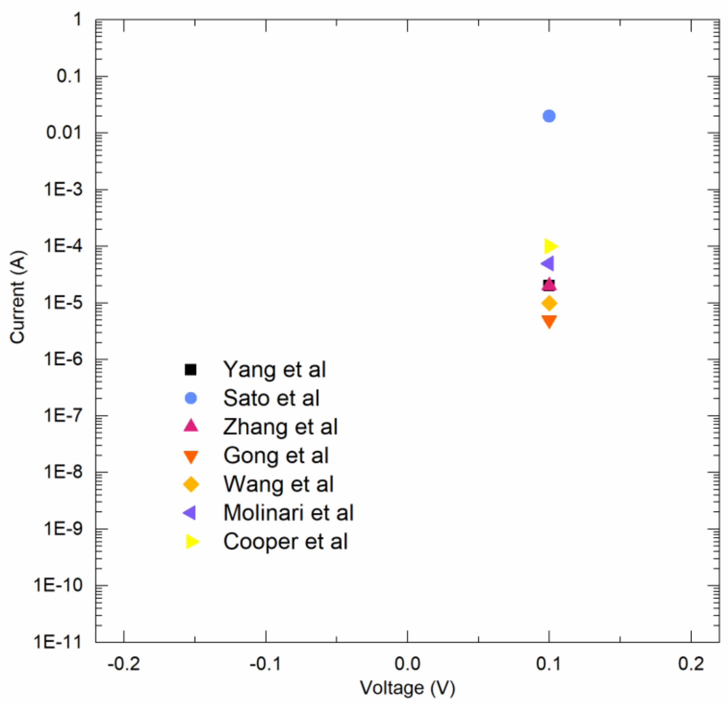

Fig. S4. Comparison of current values in function of applied voltage with the corresponding references obtained from literature. Various works have been published in recent years in the field of *in situ* TEM biasing. In this plot, data points were extracted from the reported articles at 0.1 V, showing that all current levels are in the range of 10^{-5} A [7,8,10,14,16–18]. Despite that the investigated devices in the cited contributions are not related to each other, all of them are two-terminal devices and have in common that their respective TEM *lamellae* were electrically contact using ion beam induced deposition (IBID) paths in the FIB.

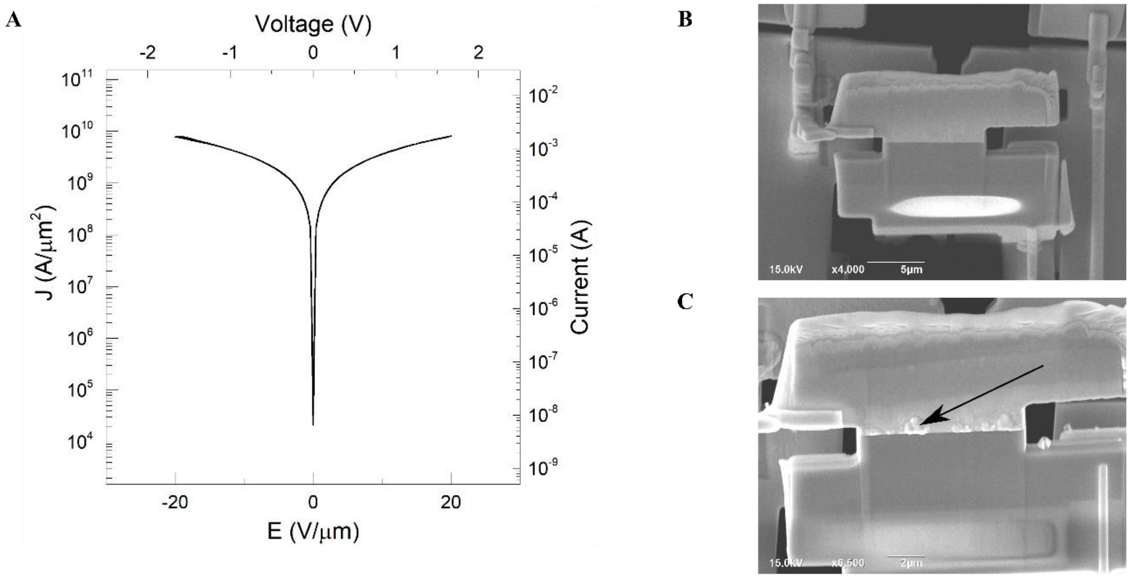

Fig. S5. Electric response and failure of a short-circuited TEM *lamella* device of memristor. **(A)** J-E curve obtained from the device (a1) prepared conventionally (sample in short-circuit). Note the high current density values of 10^{10} A/µm^2 that are achieved at 20 V/µm. Because of the short-circuit, no resistive switching behavior could be obtained from the device, on the contrary, electromigration due to joule heating on the top electrode was generated, leading to the failure of the device. **(B)** SEM image of the TEM sample prior to biasing. **(C)** SEM image of the TEM sample after biasing. Note the visible effects on the top electrode (black arrow) due to the high current densities achieved, leading to the failure of the device.

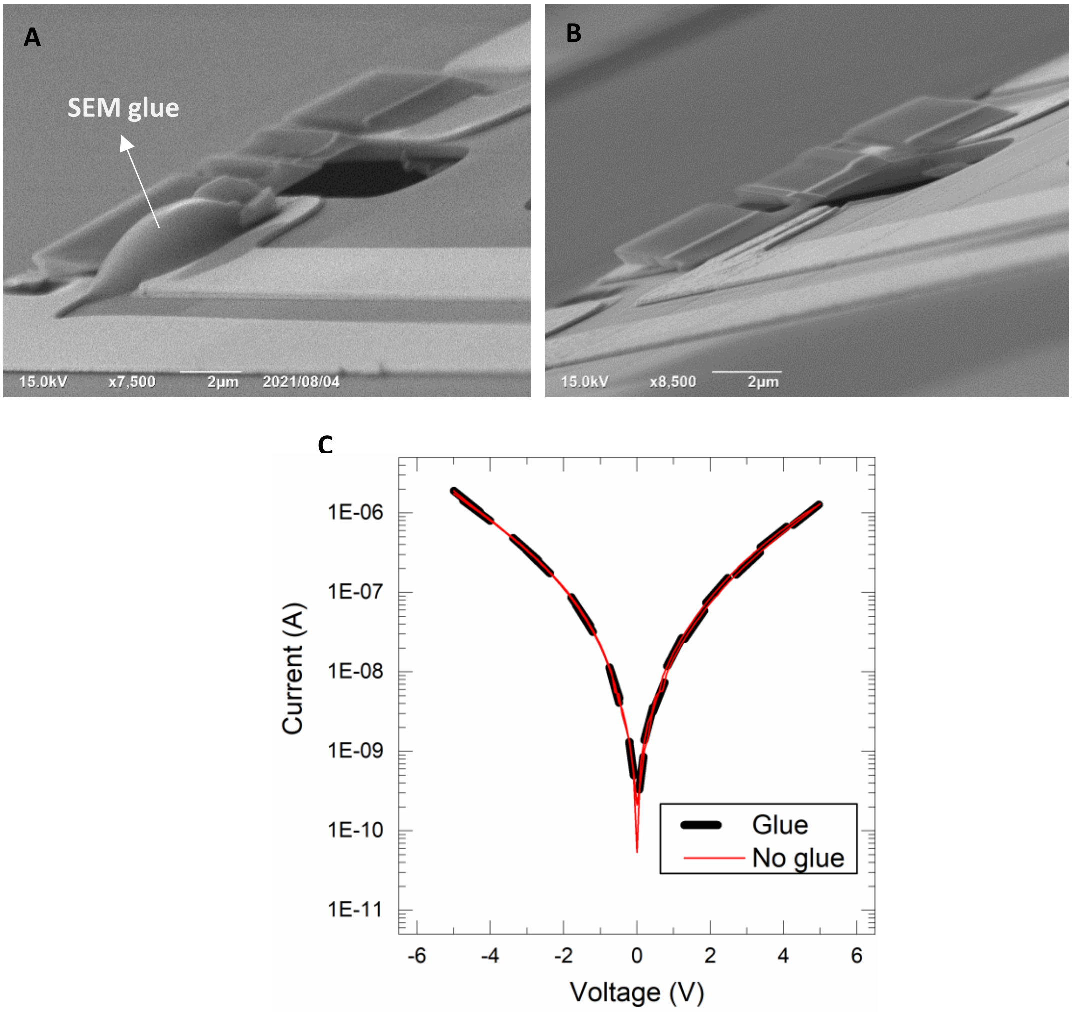

Fig. S6. TEM *lamellae* contacted with and without SEM glue. (**A**) SEM glue could be used to reinforce a TEM *lamella* device over the MEMS chip. (**B)** The *lamellae* are attached due to van der Wall forces to the MEMS-chip surface; hence, the use of glue is not a must in the present fabrication routine, but it could help other users if need it. (**C**) Electric response of a TEM *lamella* device with glue and without glue. The IV curve was initially obtained from a *lamella* without glue, then the glue was placed over the *lamella* to get the second curve. Note, the IV curves perfectly match one with another, therefore no change in the electrical response was measured.

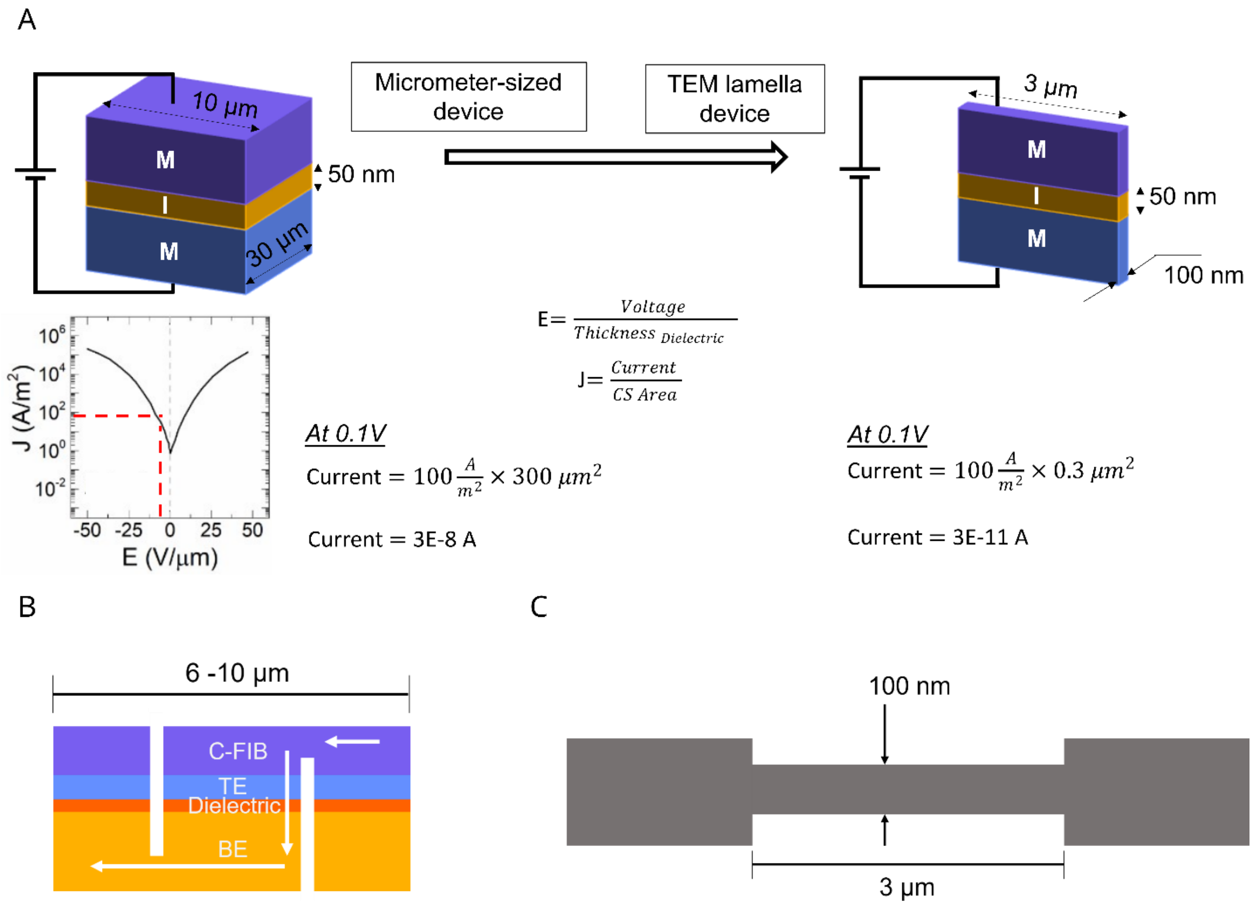

Fig. S7. Estimation of the current levels expected in a TEM *lamella* device. (A) Comparison between the leakage current obtained in a micrometer sized MIM device and the expected current values in the corresponding TEM *lamella* of the same device considering its dimensions. **(B)** Top view sketch of a TEM *lamella* prepared by our approach. Note that the white arrows described the current flow path along the stack device. **(C)** Front view of the *lamella*. The cross-section area to obtain the current density value of the electron transparent region is considered as a constant for all the prepared samples in this work, resulting in $3x10^{-13}$ m^2. A few nanometers variance in thickness might happen depending on the last FIB polishing steps, nevertheless, this will not affect the order of magnitude of the current density.

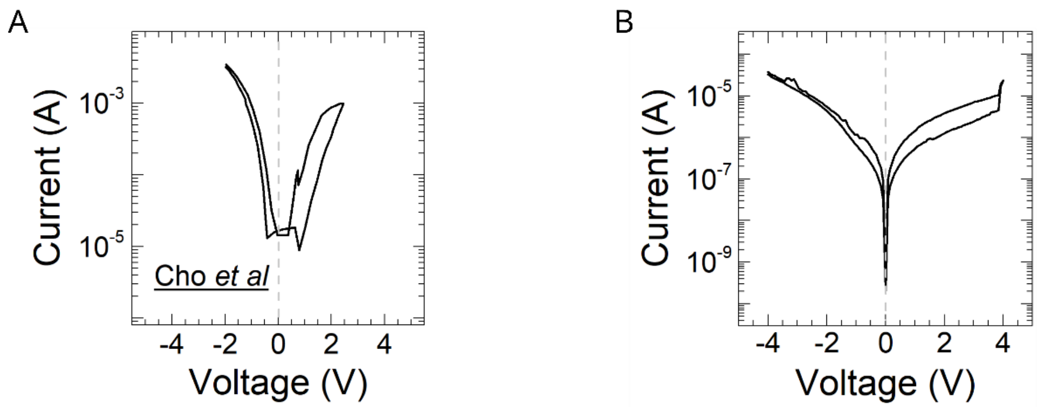

Fig. S8. Memristor a2. A self-assembled oxide film that allows the control of the resistive switching by its columnar ionic and electronic channels [22], was tested. Notice the similarities in the set/reset mechanism observed in both, (**A**) the macro device and (**B**) the corresponding FIB-based TEM *lamella* prepared by the approach described in this work.

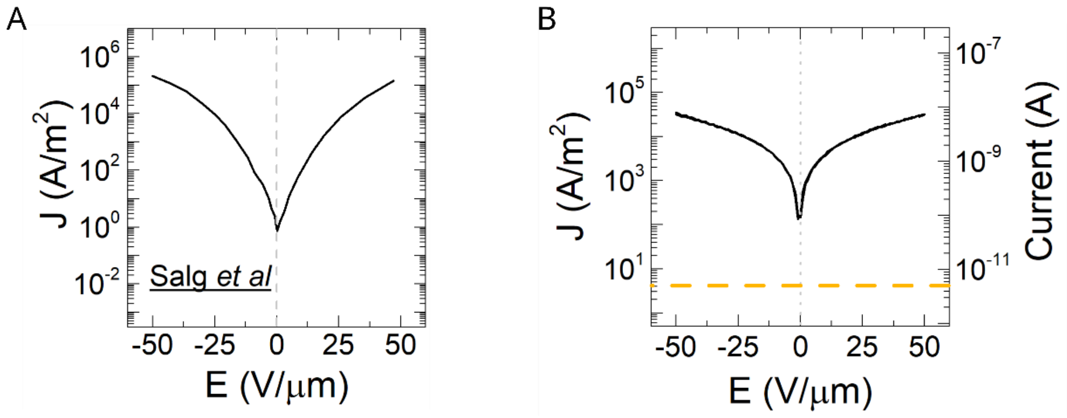

Fig. S9. Varactor b1. BST ferroelectric varactor tunable at low voltages. (**A**) Leakage current densities in the range of ~10^5 A/m^2 at 50 V/μm are commonly measured in such a macro device[24]. (**B**) In the same way, the TEM lamella counterpart replicates the trend and the current density values of the macro device.

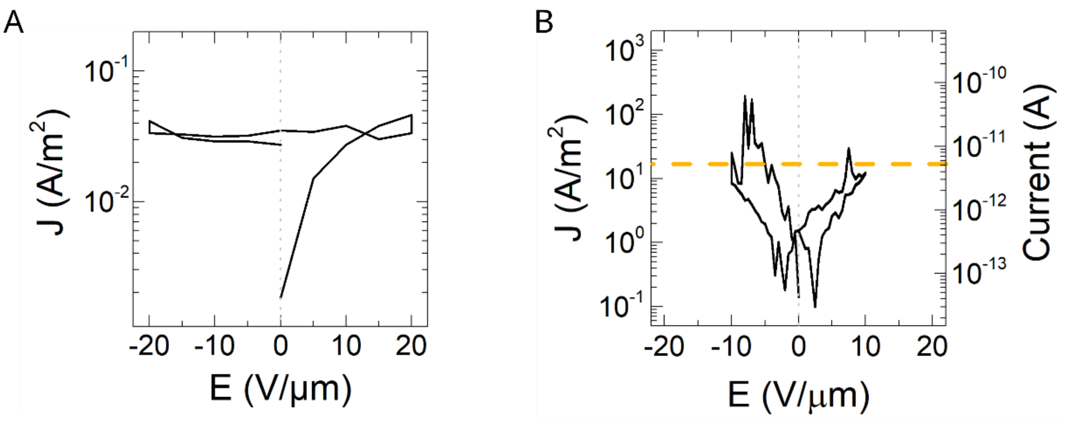

Fig. S10. Varactor b2. (**A**) Mn-doped BST ferroelectric macro device varactor with remarkably low leakage current densities (~$3x10^{-2}$ A/m^2 at 10 V/μm). (**B**) Sweep bias of the corresponding TEM lamella device yielding currents that are lower even than that of the measured in an empty MEMS-based chip (refer to Fig. S12). Therefore, in this case, the current measurement of the TEM lamella is overshadowed by the threshold current of the empty MEMS chip (yellow dash line).

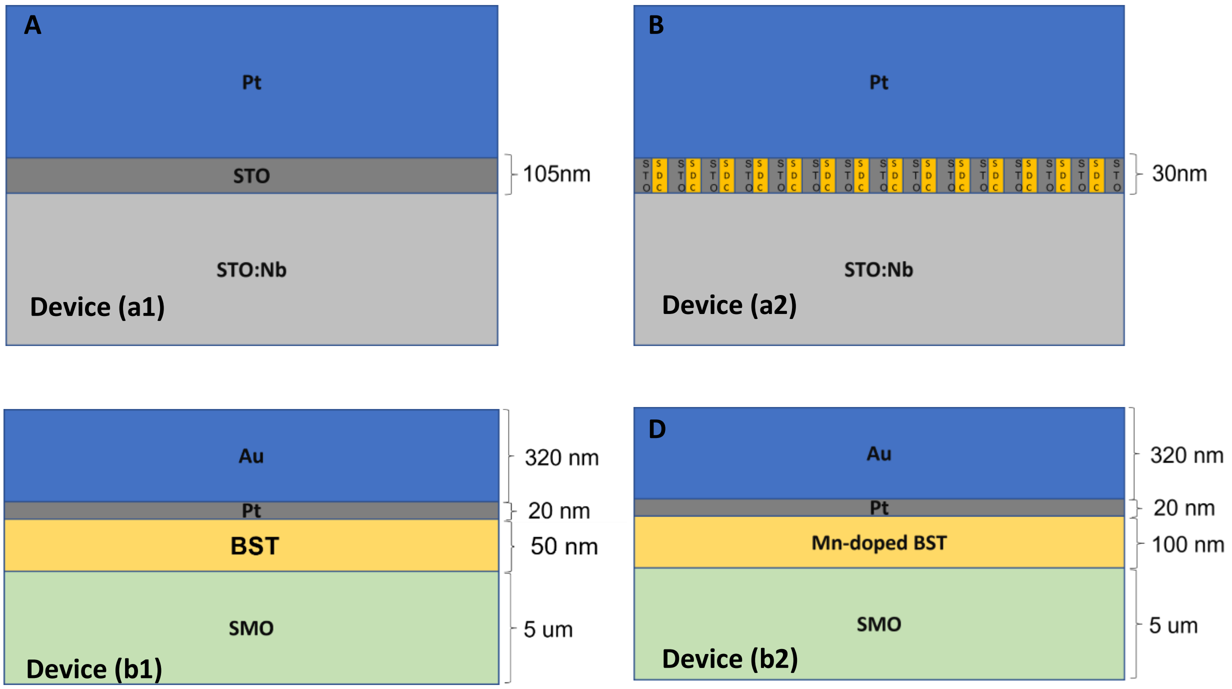

Fig. S11. Sketches of the devices shown in Table S1. (**A**) MIM device (a1) with Pt as top electrode, Nb-doped STO as substrate/bottom electrode and an intermediate layer of STO. (**B**) Memristor device (a2) of ionic and electronic channels of Sm-doped CeO_2 (SDC) STO (STO) grown on a substrate/bottom electrode of Nb-doped $SrTiO_3$ and Pt as top electrode. (**C**) Varactor device (b1) of $BaSrTiO_3$ (BST) as the dielectric layer. (**D**) Low leakage (b2) varactor device with Mn-doped BST as the intermediate layer. Both, (C and D) have a Au/Pt top electrode and the bottom electrode is a highly conductive $SrMoO_3$ [23] .

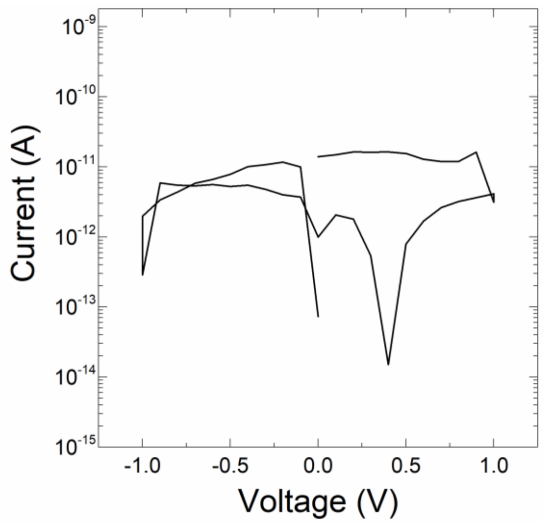

Fig. S12. Current generated by an empty MEMS-based chip. A pristine MEMS chip without any TEM *lamella* on it has been electrically tested. As seen, the chip itself generates currents in the range of 5×10^{-12} A on average. Therefore, the values near 0 V of a contacted TEM *lamella* or low leakage current devices that produce currents in the same or lower magnitude of that of an empty MEMS-based chip will be influenced by this parasitic current.

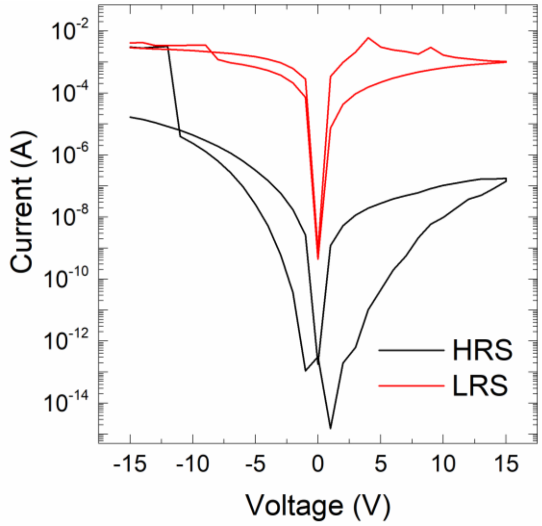

Fig. S13. Resistive switching behavior of the Pt/STO/Nb:STO macro device. Electric response of (a1) millimeter-size device from where the TEM *lamellae* has been extracted. Note the set of the device obtained from HRS (black curve) to LRS (red curve) at around -10 V.

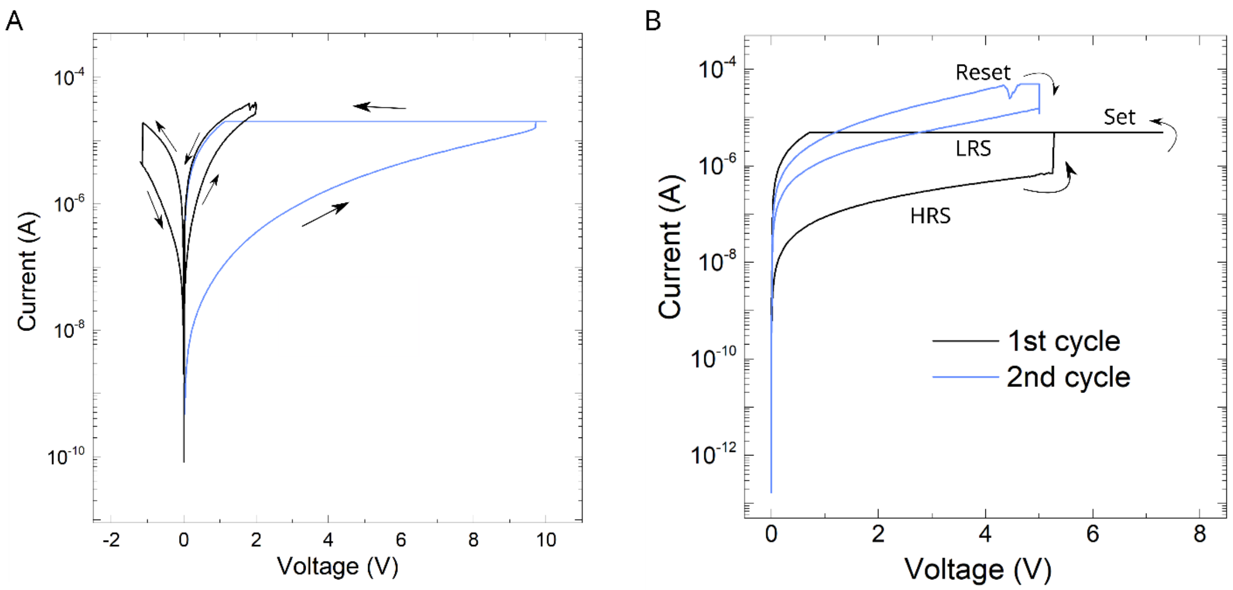

Fig. S14. Measurement of resistive switching behavior of TEM *lamella* devices of (a1) and (a2) inside the TEM. (**A**) Bipolar resistive switching obtained in memristor a1 inside the microscope. Note that the forming process as well as cycling (set/reset) of the device has been successfully achieved in TEM *lamellae* prepared by our FIB-based sample preparation routine. (**B**) Electrical response of TEM *lamella* device of memristor (a2) clearly showing set/reset mechanisms in a unipolar resistive switching process.

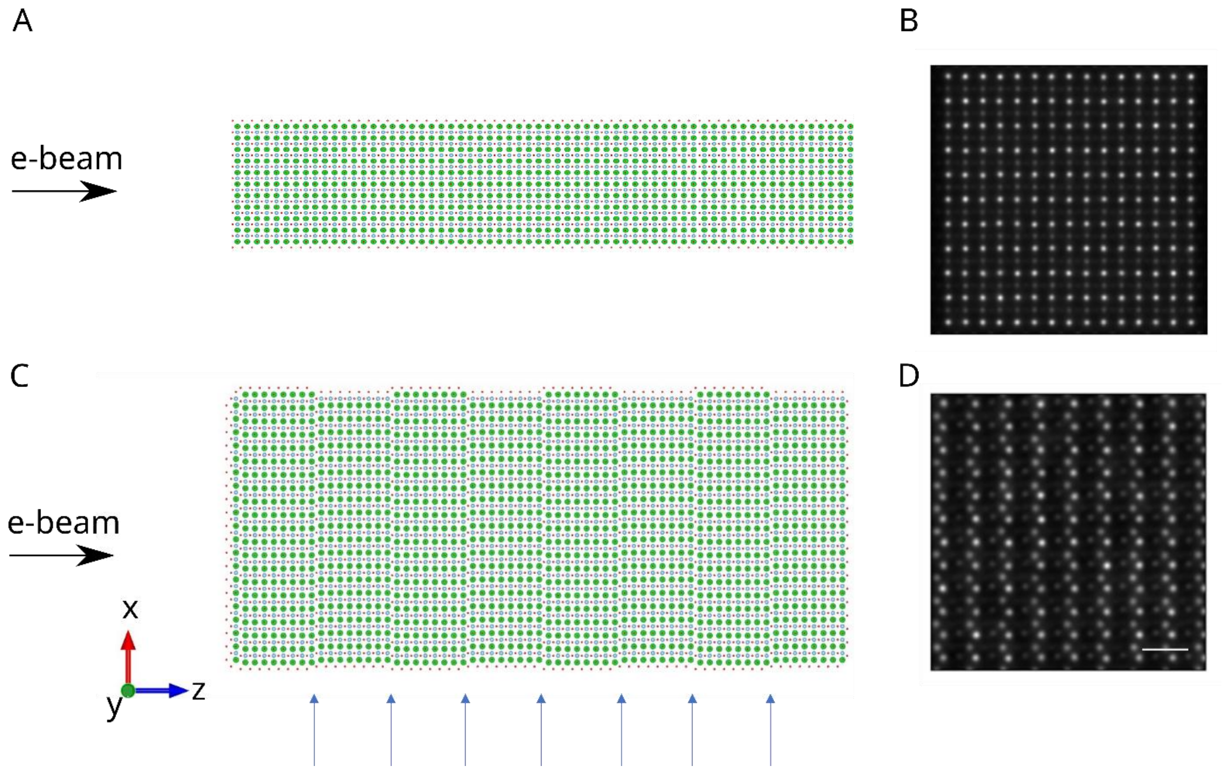

Fig. S15. Structural model and simulations of STO before and after resistive switching. (A) Visualization of $SrTiO_3$ along the [001] ZA in LRS. (**B**) Corresponding Multislice simulation along the [110] ZA, note the e-beam viewing direction in (A). (**C**) Visualization of extended defects along the [001] zone axis in HRS. Note the slip-plane features that are highlighted with the blue arrows. (**D**) Corresponding Multislice simulation along the [110] ZA, note the e-beam viewing direction in (C). The scale bar is 0.5 nm.

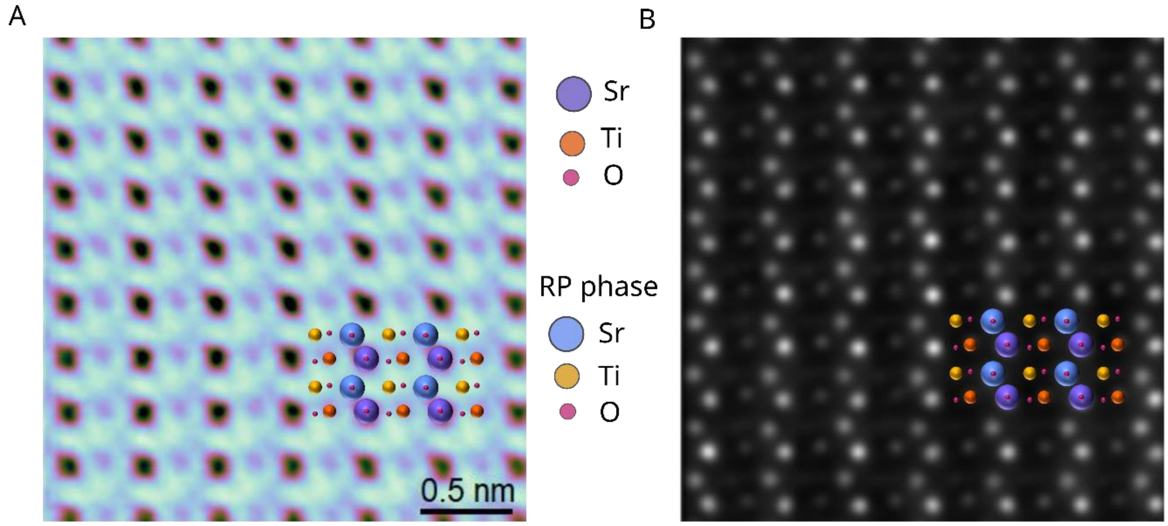

Fig. S16. Extended defect HAADF-STEM image and simulation viewed along the [110] ZA in the active STO layer of device (a1). (A) Experimental inverse color coded HAADF-STEM image to enhance visualization. **(B)** Corresponding simulated HAADF-STEM image generated with the Multislice algorithm corresponding to the image shown in (A). Note the perfect agreement between the image and the simulation, also refer to the model system in the insets.

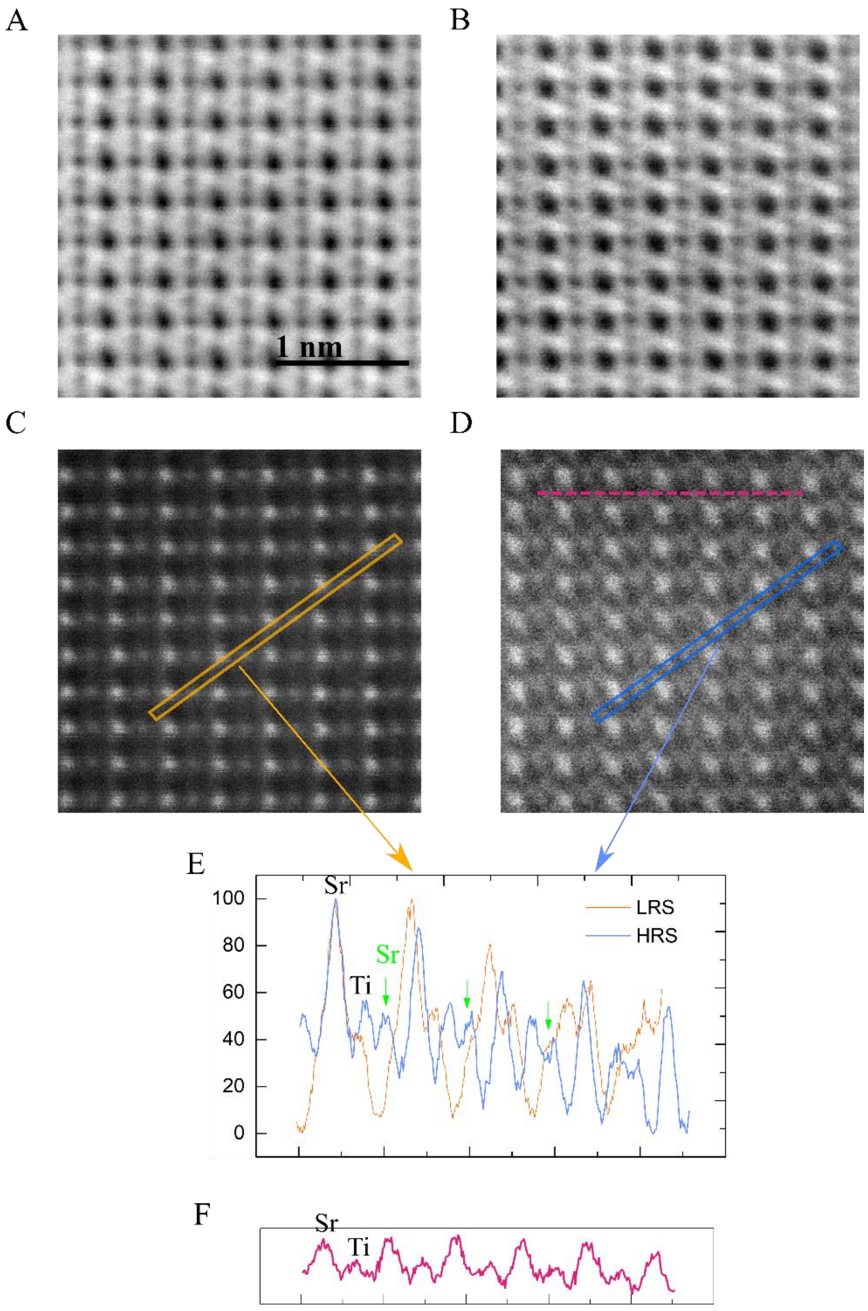

Fig. S17. HAADF-STEM and ABF-STEM images of the active STO layer in memristor (a1) corresponding to Fig. 3. (A and **B)** ABF-STEM images prior to and after resistive switching, respectively. (**C** and **D**) HAADF-STEM images prior to and after resistive switching, respectively. (**E)** Normalized intensity profile comparing the position where the extended defect is formed after resistive switching in HRS based on the model of Fig. 3. Note in LRS (C) there are no visible intensity peaks between Sr or Ti atom columns (orange curve), while in HRS (D) the peaks (green arrows) corresponding to Sr atom columns of the slip plane are observed (blue curve). (**F**) Intensity profile of the red curve in (D), note the Ti atom column, next to the Sr atom column corresponding to the slip plane.

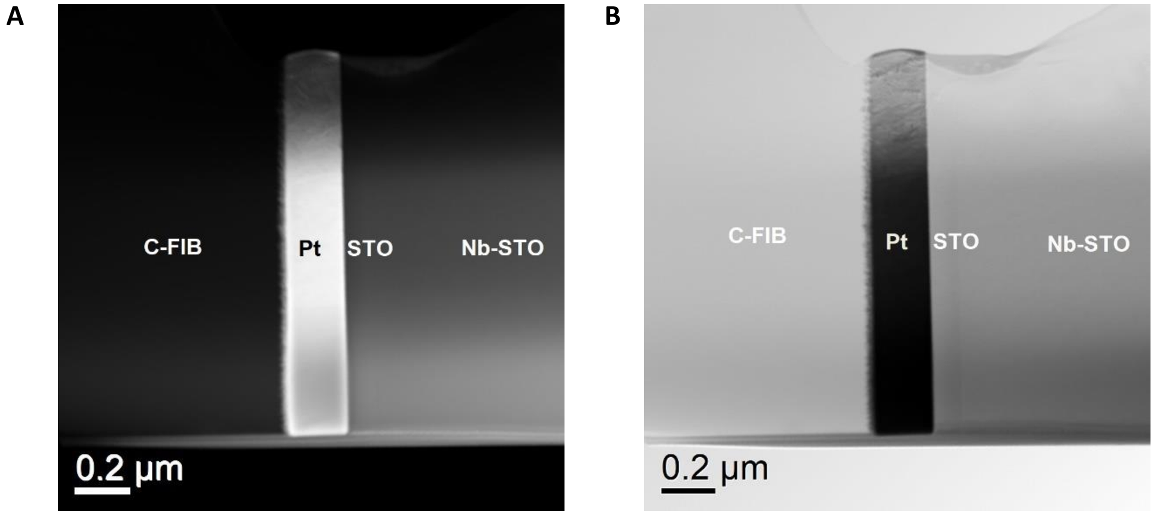

Fig. S18. HAADF and BF-STEM images of device (a1) corresponding to Fig. 4A. The images were obtained while scanning the electron beam to measure the e-beam effect on the electrical response of the TEM *lamella* device **(A)** HAADF-STEM. (**B**) BF-STEM image.

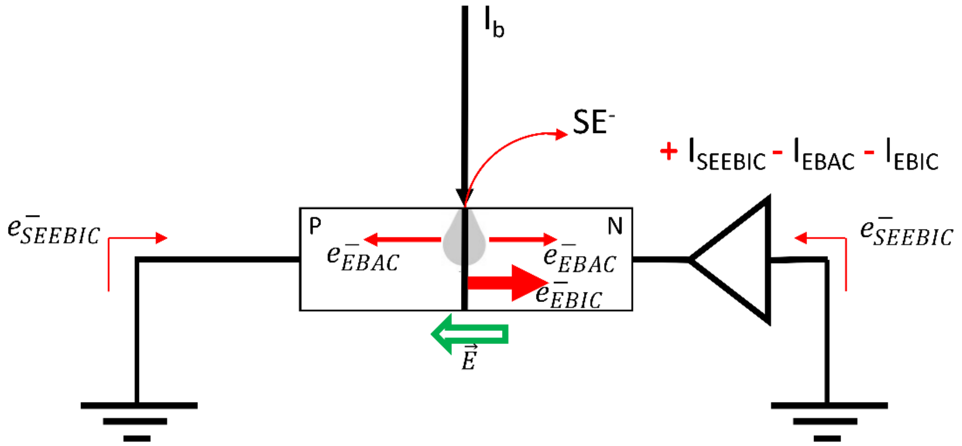

Fig. S19. Sketch of electron beam induced currents (EBIC) in STEM mode. Three kinds of induced currents can be generated in a TEM *lamella*. Electric-field related (refer as I_{EBIC} in the sketch), absorbed current (I_{EBAC} in the sketch), and secondary emission-related current (I_{SEEBIC} in the sketch). Note that the current flow direction is represented by the red arrows in the setup schematic. I_{EBIC} can be considered negligible because no electron-hole generation is relevant in our device at 0 V. I_{EBAC} can also be neglected due to sample thickness, however depending on the material density, this current might exist. Therefore, I_{SEEBIC} is the main induced current mechanism that can be considered here.

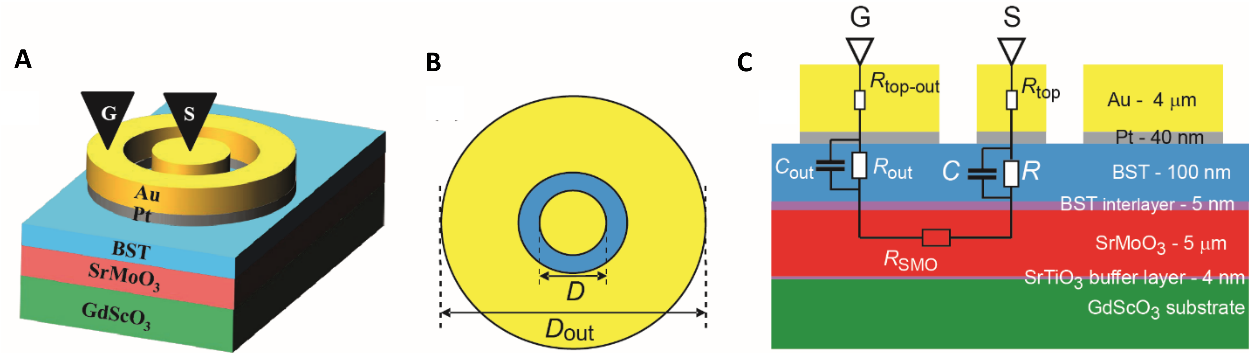

Fig. S20. Schematic of the macro devices (b1), and (b2). (A) 3D sketch, **(B)** top-view sketch, and **(C)** cross-section view of the MIM test structures. For measurements of the leakage current, a bias voltage was applied between the signal (S) and ground (G) pads with a diameter of D=20-60 µm and D_{out}=350 µm, respectively. The thicknesses of the thin layers and the equivalent circuit for the measurements of the leakage current are shown in (c). For the produced test structures, $D_{out} \gg D$ and, therefore, $R_{out} \ll R$. Moreover, $R_{top-out}, R_{top}, R_{SMO} \ll R$. Thus, the measured leakage current is dominated by the leakage current in the small central "Signal" (S) MIM element [23–25].

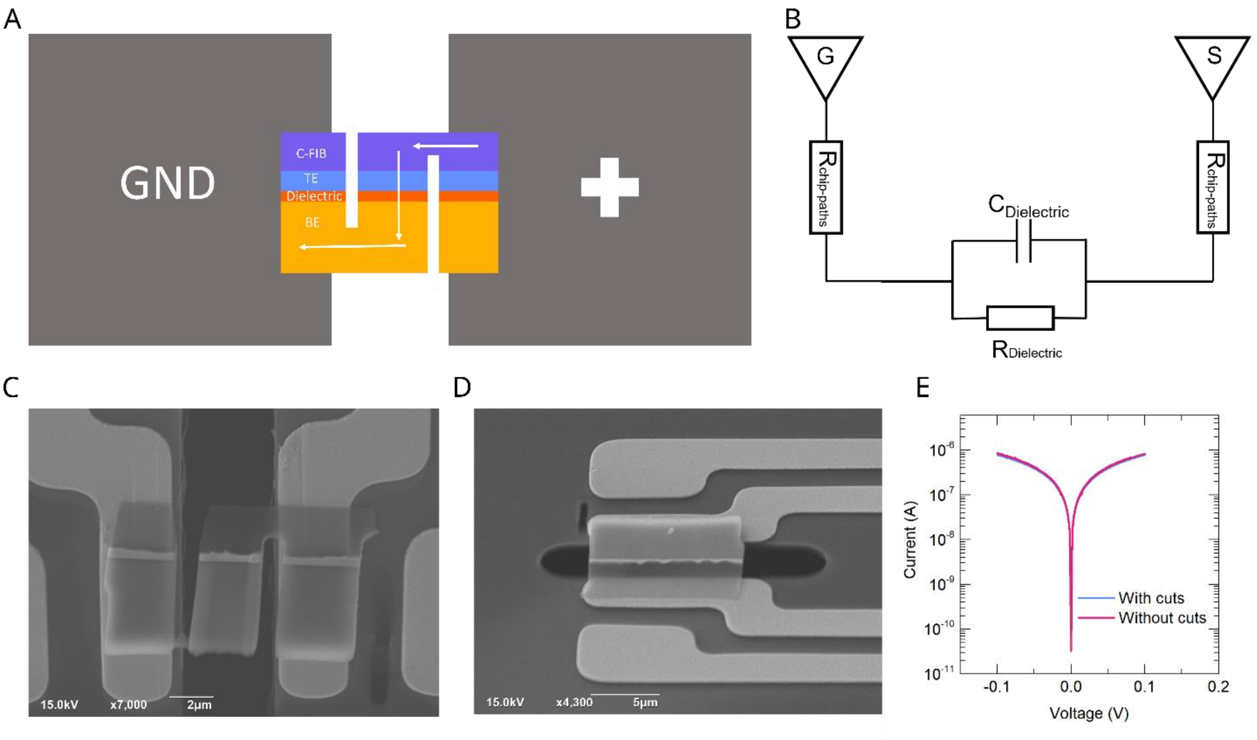

Fig. S21. Sketch of the electrical circuit of a TEM *lamella* device and the influence of lateral FIB cuts. (**A**) In this configuration, the positive bias electrode is applied to the right lead of the MEMS chip while the left electrode/lead is grounded (GND). The generated current flow (arrow) is limited by the lateral FIB cuts, regardless of where the TEM sample is contacted on its sides. Therefore, the current is forced to flow across the electron transparent region of interest (ROI) (white line); from the top electrode (TE) to the bottom electrode (BE). (**B**) The equivalent circuit structure of the TEM *lamella* test structure. The resistance of the chip leads ($R_{chip\text{-}paths}$) is considered negligible. (**C**) SEM view of the *lamella* represented in (A). (**D**) TEM *lamella* without cuts, notice that the entire top and bottom electrodes of the device are placed independently on the leads of the MEMS-based chip, avoiding the cuts described in (A). (**E**) I-V curves of *lamellas* obtained from a high-leakage device with and without lateral cuts, both curves showed the same trend and current range. Therefore, the cuts to limit the current flow do not influence the electrical response of the device. The TEM sample shown in (D) could be considered ideal compared with the one in (A), but the attachment of the TEM *lamella* to the chip is still very challenging. However, as previously indicated in (E), the effect on the electrical behavior is insignificant.

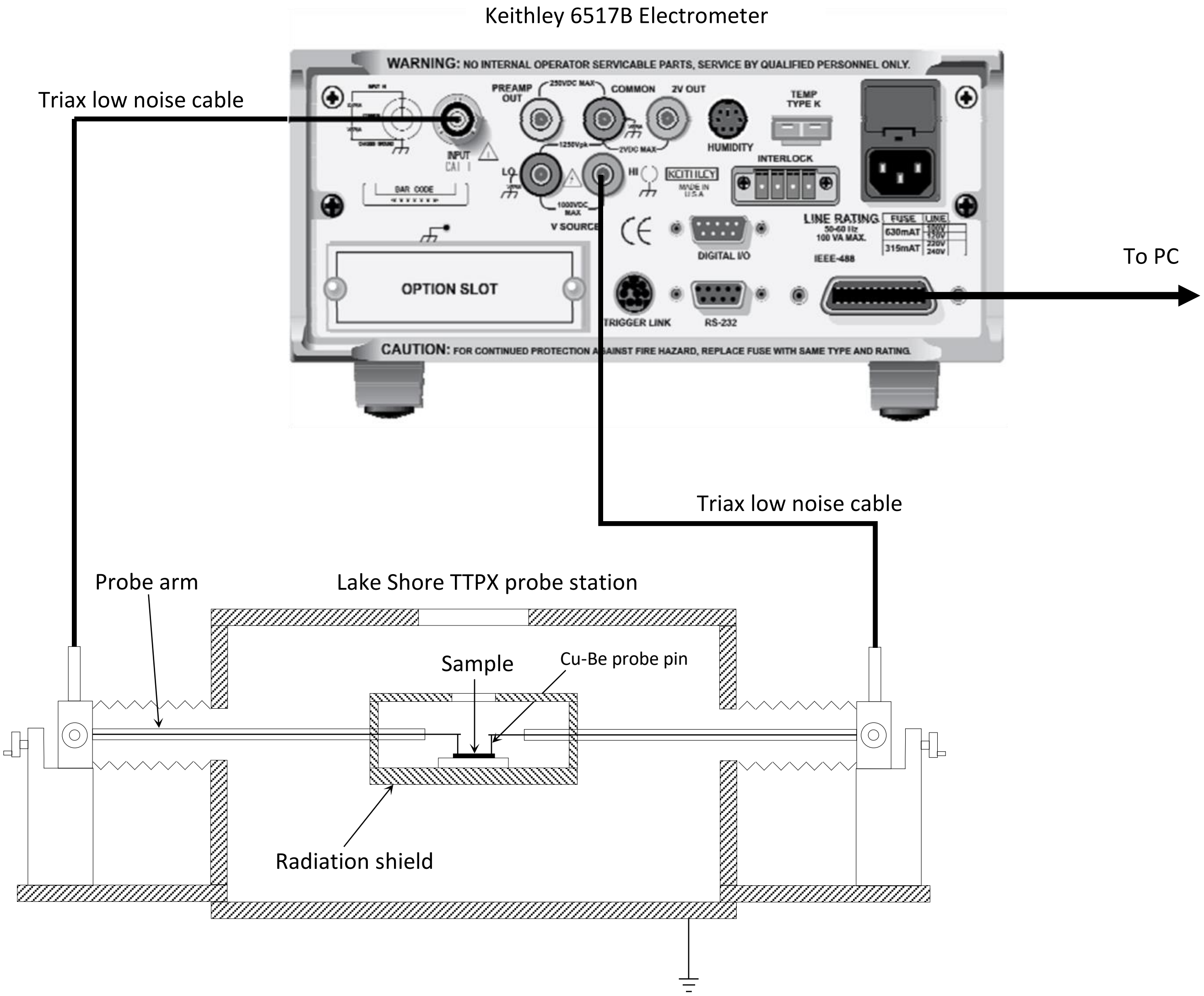

Fig. S22. Schematic of the experimental setup used to measure leakage currents in high-resistive devices and the empty "Lightning" MEMS-based chip by DENSsolutions. Two pads of the "Lightning" chip are connected, using Cu-Be pins of the Lake Shore TTPX probe station. The Keithley 6517B electrometer applies a voltage at the output "V-source Hi" and measures the current at the "INPUT Hi". The "V-source Lo" is internally connected to "INPUT Lo". The measurements are controlled by a PC via RS232 interface.

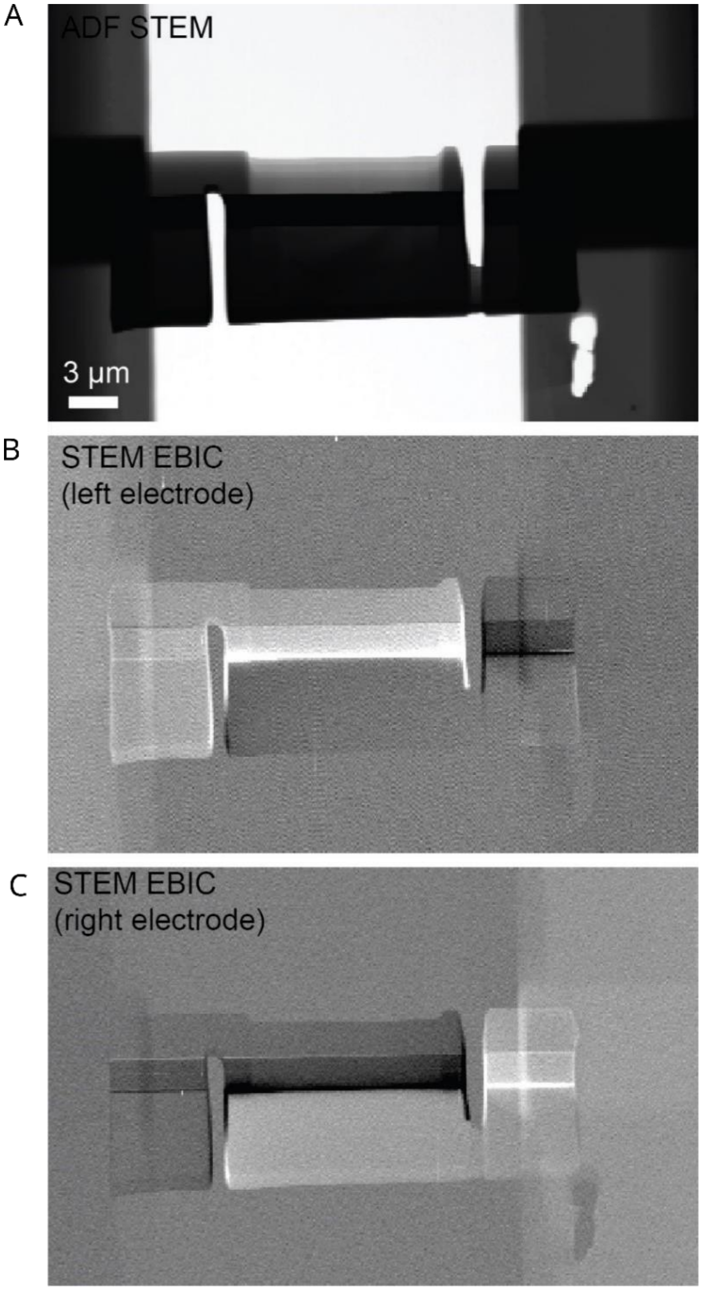

Fig. S23. Low magnification STEM EBIC acquired using a two-channel STEM EBIC system from NanoElectronic Imaging, Inc (NEI). (**A**) Annular Dark Field (ADF) STEM image. (**B**) STEM EBIC of the left-hand side chip lead (top electrode of the device). (**C**) STEM EBIC of the right-hand side chip lead (bottom electrode of the device). Note the similar contrast achieved between the left (right) side hand of the TEM *lamella* device and the top (bottom) electrode in the STEM EBIC images, implying a relatively low resistance due to lamella attachment. Note that SE emission is always stronger on surfaces parallel to the beam "edge effect", which is what causes the "sharp contrast" at the edges of the lamella. However, along the surface of the TEM lamella the leakage is practically eliminated, this means, the dielectric remains insulating overall.
